# Advanced progress of vestibular compensation in vestibular neural networks

**DOI:** 10.1111/cns.70037

**Published:** 2024-09-13

**Authors:** Jun Wang, Yuejin Zhang, Huajing Yang, E. Tian, Zhaoqi Guo, Jingyu Chen, Caijuan Qiao, Hongqun Jiang, Jiaqi Guo, Zhanghong Zhou, Qing Luo, Shiyu Shi, Hongyi Yao, Yisheng Lu, Sulin Zhang

**Affiliations:** ^1^ Department of Otorhinolaryngology, Head and Neck Surgery The First Affiliated Hospital, Jiangxi Medical College, Nanchang University Nanchang China; ^2^ National Clinical Research Center for Otolaryngologic Diseases Jiangxi Branch Center Nanchang China; ^3^ Department of Otorhinolaryngology Union Hospital, Tongji Medical College, Huazhong University of Science and Technology Wuhan China; ^4^ Institute of Otorhinolaryngology Union Hospital, Tongji Medical College, Huazhong University of Science and Technology Wuhan China; ^5^ Department of Physiology, School of Basic Medicine Huazhong University of Science and Technology Wuhan China; ^6^ Department of Ophthalmology Tongji Hospital, Tongji Medical College, Huazhong University of Science and Technology Wuhan China; ^7^ Department of Rehabilitation Liyuan Hospital of Tongji Medical College, Huazhong University of Science and Technology Wuhan China

**Keywords:** commissural inhibition system, medial vestibular nuclei, neural networks, neurotransmitters, vestibular compensation

## Abstract

Vestibular compensation is the natural process of recovery that occurs with acute peripheral vestibular lesion. Here, we summarize the current understanding of the mechanisms underlying vestibular compensation, focusing on the role of the medial vestibular nucleus (MVN), the central hub of the vestibular system, and its associated neural networks. The disruption of neural activity balance between the bilateral MVNs underlies the vestibular symptoms after unilateral vestibular damage, and this balance disruption can be partially reversed by the mutual inhibitory projections between the bilateral MVNs, and their top‐down regulation by other brain regions via different neurotransmitters. However, the detailed mechanism of how MVN is involved in vestibular compensation and regulated remains largely unknown. A deeper understanding of the vestibular neural network and the neurotransmitter systems involved in vestibular compensation holds promise for improving treatment outcomes and developing more effective interventions for vestibular disorders.

## Graphical abstract

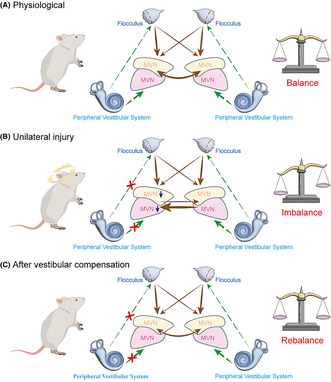

## 1 INTRODUCTION

Vestibular disorders are prevalent conditions that arise from various factors, including stroke, head trauma, and aging, leading to postural imbalance, gaze instability, spatial orientation difficulties, and vertigo, profoundly impacting the overall quality of life.^1^, ^2^, ^3^, ^4^ Interestingly, these symptoms caused by unilateral vestibular injury can partially alleviate over time through central nervous system (CNS) plasticity, called vestibular compensation,^5^, ^6^, ^7^, ^8^, ^9^, ^10^ which can be promoted by medications and vestibular rehabilitation. The two medial vestibular nuclei (MVN) on either side of the brainstem are considered the hub of the vestibular system and the critical brain region for vestibular compensation.^10^ They are mutually projected to each other via GABAergic and glycinergic neurons,^11^ integrating the peripheral vestibular input and top‐down regulation from other brain regions such as the cortex,^10^ cerebellum,^12^ thalamus and hypothalamus.^13^, ^14^

**FIGURE 1:**
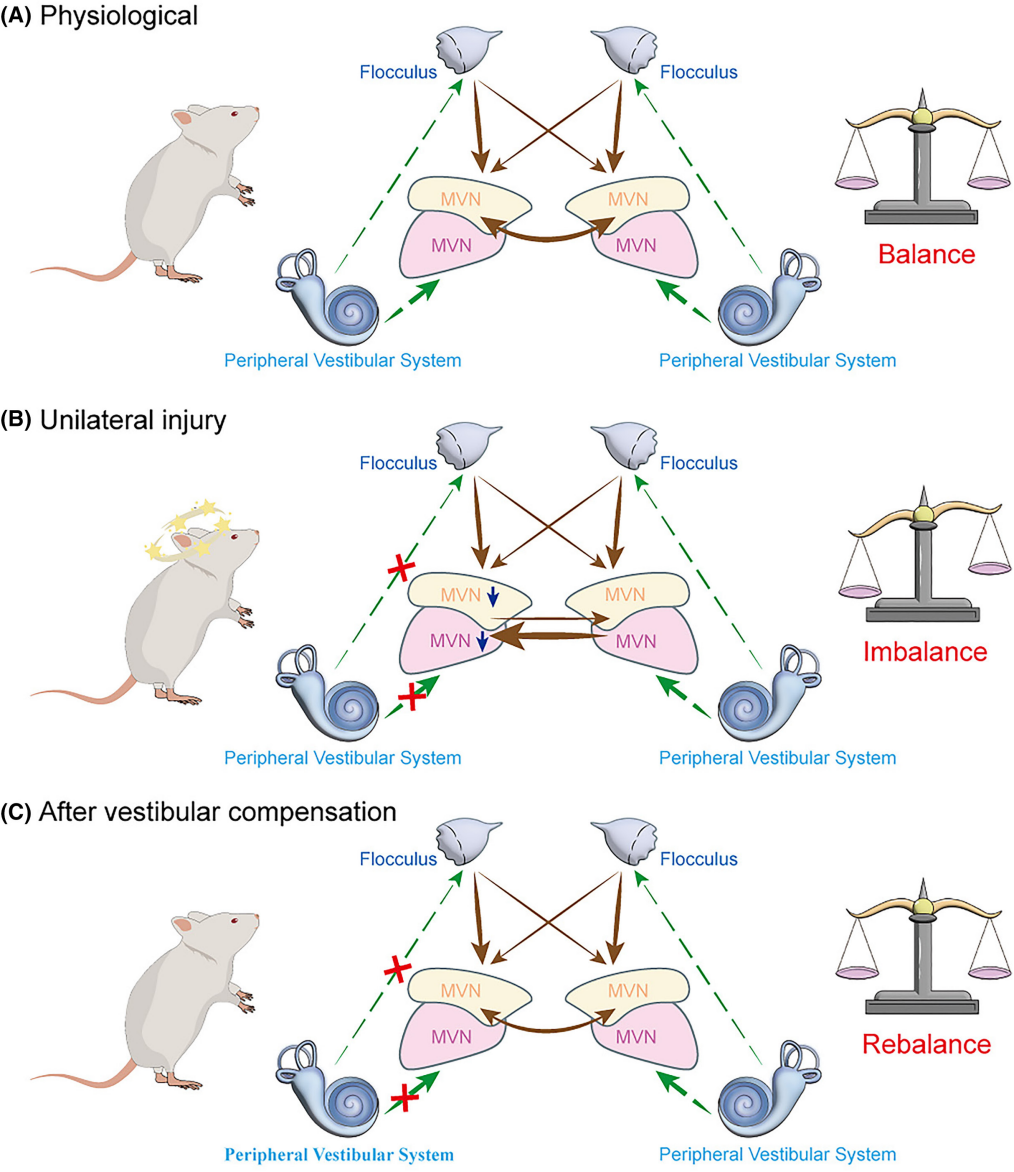
The mechanism of vestibular compensation.^11^ The MVN receive afferent signals from the peripheral vestibular system and other brain regions, such as the cerebellum, the neuronal activity is balanced between the bilateral vestibular nuclei. After the unilateral peripheral injury, this loss of afferent leads to down‐regulation of neuronal activity in the ipsilateral vestibular nucleus, while up‐regulation in the contralateral side. This imbalance is believed leading to the symptoms of vestibular disorders. CNS can partially rebalance the neural activity between bilateral MVN, and reverse the vestibular symptoms. In contrast to the ipsilateral side, the contralateral MVN neurons become more active, which might be due to the reduced inhibitory input from the ipsilateral vestibular nucleus. Besides the inhibitory commissural projections between the bilateral MVN, the cerebellum and other brain region inputs are also critical for vestibular compensation. Green dashed line with arrowheads: Excitatory transmission; Brown dashed line with arrowheads: Inhibitory transmission. CNS, Central nervous system; MVN, Medial Vestibular Nucleus.

Following unilateral vestibular damage, neurons in the ipsilateral vestibular nucleus become sedate due to reduced input from the vestibular system; in contrast, neurons in the contralateral vestibular nucleus become more active, which might be due to the reduced inhibitory input from the ipsilateral vestibular nucleus (Figure 1).^11^ This activity imbalance between both sides of the vestibular nucleus is believed to be underlying the vestibular syndromes,^15^ which can be partially reversed during vestibular compensation.^16^, ^17^ Understanding this endogenous recovery mechanism will be beneficial for developing new strategies for vestibular disorder treatment. It will also be fruitful for the investigation of CNS postlesional plasticity, the ability of CNS to compensate for lesion‐impaired functions, including movement, cognition, and behavior. In this review, we will focus on the vestibular compensation mechanism of MVN and its related regulation neural networks to address the question: after unilateral vestibular injury, how does MVN integrate signals from the whole brain neural network and regulate vestibular compensation to facilitate recovery?

## 2 THE STRUCTURE AND FUNCTION OF THE VESTIBULAR NUCLEI

The vestibular nuclei are located in the brainstem, the lateral walls of the fourth ventricle, extending towards the junction of the medulla and the cerebellum.^18^, ^19^ The vestibular nuclei receive most of the peripheral vestibular system projection to the CNS, and integrate multiple sensory inputs, orchestrating brain regions to control visual fixation, balance, and spatial orientation.^20^, ^21^ Each side of the vestibular nucleus is composed of the superior, spinal, medial, and lateral vestibular nucleus^18^, ^19^; among these subnuclei, MVN is the largest and most widely studied region,^22^ which has different functions longitudinally, caudal MVN neurons are the input source to ipsilateral flocculus, while the rostral MVN receives input from ipsilateral flocculus Purkinje cells.^22^

## 3 THE CELL TYPES OF THE VESTIBULAR NUCLEI

According to cell size, vestibular nuclei cells can be divided into magnocellular and parvocellular cells. The magnocellular cells are located in the external region of vestibular nuclei and are mainly composed of multipolar glutaminergic neurons. The parvocellular cells, on the other hand, are mainly bipolar GABAergic neurons and are located near the fourth ventricle.^23^, ^24^, ^25^ GABAergic neurons in vestibular nuclei were divided into five groups^26^: The first group of GABAergic neurons forms associational fibers between the bilateral vestibular nuclei. The second, third, and fourth groups of GABAergic neurons project to the oculomotor nuclei, the inferior olive nucleus, and the spinal motor neurons, respectively. The fifth group is comprised of local interneurons in the MVN.

## 4 THE EXCITABILITY OF VESTIBULAR NUCLEI NEURONS DURING VESTIBULAR COMPENSATION

After the unilateral vestibular injury, glutamate concentration decreased during the first 2 days on the ipsilateral side of MVN,^27^ consistent with the observation that the primary vestibular fibers gradually degrade due to the deafferentation peripheral vestibular system.^28^ Nevertheless, the heightened frequency of excitatory postsynaptic currents (EPSC) implies an augmented discharge from excitatory projections from other brain regions, which might promote the restoration of ipsilateral MVN excitability and the neural activity balance of the bilateral MVN.^29^

On the contrary, the quantity of GABAergic terminals increased within the first 3 days after injury; in consistency, the inhibitory postsynaptic currents (IPSC) increased in the ipsilateral MVN.^30^, ^31^ This enhanced inhibitory input may originate from GABAergic neurons in the contralateral vestibular nucleus^31^ and local GABAergic neurons in the ipsilateral MVN.^32^, ^33^ Three days after the unilateral vestibular injury, IPSC frequency gradually declines, accompanied by an increase in the frequency of EPSCs, restoring the neural activity balance in both vestibular nuclei.^34^

The rebalancing of firing activity in neurons of bilateral MVN is a result of the combined effect of these excitatory and inhibitory events, while the regulation of inhibitory transmission may be dominant.^10^

## 5 COMMISSURAL INHIBITORY SYSTEM

The projections between the bilateral vestibular nuclei are mainly inhibitory GABAergic transmission, mediated by GABA_A_ receptors rather than GABA_B_ receptors, constituting the commissural inhibitory system.^15^ When the unilateral vestibular is damaged, the reduced neural activity of the ipsilateral MVN results from both the loss of peripheral vestibular input and the contralateral MVN innervation through the commissural inhibition system.^11^, ^32^, ^35^ This long‐distance inhibitory projection from the contralateral vestibular nucleus likely plays a pivotal role,^10^ as fewer MVN neurons are silenced, and the firing frequency of neurons is less reduced after bilateral peripheral damage, compared to unilateral damage. Importantly, no neural activity imbalance is observed in the bilateral vestibular nucleus, and the animals did not display any asymmetric postural behavior after bilateral vestibular damage.^36^ Consistent with these observations, although GABA levels increased in the ipsilateral vestibular nucleus, several days after unilateral vestibular damage, GABA levels returned to normal.^32^ In the contralateral vestibular nucleus, GABA levels decreased significantly,^37^ and this rebalance of commissural inhibition is synchronized with the recovery of behavior.^32^ All the above findings collectively suggest that the commissural inhibitory system is critical in vestibular compensation.

## 6 VESTIBULAR NEURAL NETWORK AND NEUROTRANSMITTERS DURING THE VESTIBULAR COMPENSATION

Besides the local circuitry in MVN and the commissural inhibitory system between the bilateral MVN, other brain regions also play a critical role in vestibular compensation, probably via modulating MVN neurons. Recently, significant advances have been made in elucidating the brain regions targeting MVN GABAergic and glutamatergic neurons (Table 1). More than 50 upstream nuclei were identified for MVN GABAergic and glutamatergic neurons, distributed mainly in the hindbrain, including the medulla, pons, midbrain, and cerebellum.^14^, ^38^, ^39^ Most brain regions of the medulla, pons, midbrain and cerebellum are closely related to MVN and project to GABAergic and glutamatergic neurons of MVN, these neural networks may be related to the regulation of eye movements and postural control.^40^, ^41^, ^42^ MVN GABAergic neurons received more inputs than glutamatergic neurons (Table 2) from distant brain regions, including the thalamus, hypothalamus and cortex. These inputs are related to sleep, eye movement, and postural control.^43^, ^44^, ^45^, ^46^ Inhibitory projections of the cerebellar lobules mainly project to the glutamatergic neurons of the MVN (Table 3).^47^ The functions of these upstream nuclei relate to balance maintenance, emotion control, and circadian rhythm regulation, suggesting the comorbid of vestibular disorders with anxiety/depression and sleep disorder, and the treatments aimed at these brain regions may aid in the promotion of vestibular compensation.

**TABLE 1:** The distribution of brain regions that innervate both GABAergic and glutamatergic neurons in MVN.^38^, ^39^

Brain regions	Nucleus
Medulla	Cuneate nucleus, Gigantocellular reticular nucleus, Intermediate reticular nucleus, Medullary reticular nucleus (dorsal and ventral part), Nucleus of the solitary tract, Paragigantocellular nucleus (dorsal and lateral), Parvicellular reticular nucleus, Prepositus nucleus, Raphe magnus nucleus, Spinal trigeminal nucleus
Pons	Abducens nucleus, Central gray of the pons, Deep mesencephalic nucleus, Dorsal tegmental nucleus, Ko¨lliker‐Fuse nucleus, Locus coeruleus, Parabrachial nucleus, Pontine reticular nucleus (oral and caudal part), Principal sensory trigeminal nucleus, Sub‐coeruleus nucleus, Supra‐trigeminal nucleus
Midbrain	Dorsal raphe nucleus, Interstitial nucleus of Cajal, Supra‐oculomotor periaqueductal gray, Periaqueductal gray, Pedunculopontine tegmental nucleus, Substantia nigra
Cerebellum	Vestibulo‐cerebellar nucleus, Medial (fastigial) cerebellar nucleus, Interposed cerebellar nucleus, lateral cerebellar nucleus

**TABLE 2:** The distribution of brain regions that innervate GABAergic neurons in MVN.^38^

Brain regions	Nucleus
Medulla	Facial nucleus, Hypoglossal nucleus, Linear nucleus of the medulla, Nucleus of Roller, Raphe obscurus nucleus Reticular nucleus (lateral and rostroventrolateral)
Pons	Barrington's nucleus, Median raphe nucleus, Motor trigeminal nucleus, Paramedian reticular nucleus, Reticulotegmental nucleus of the pons, Rostral periolivary region, Tegmental area (dorsomedial, laterodorsal and posterodorsal), Ventral nucleus of the lateral lemniscus
Midbrain	Anterior pretectal nucleus, External cortex of the inferior colliculus, Interpeduncular nucleus, lateral subnucleus, Nucleus of Darkschewitsch, Oculomotor nucleus, Raphe cap, Red nucleus (parvicellular part), Superior colliculus (deep gray layer and intermediate gray layer), Ventral tegmental area
Thalamus	Zona incerta
Hypothalamus	Lateral hypothalamic area, Paravaentricular hypothalamic nucleus, Posterior hypothalamic area, Prerubral field
Cortex	Primary motor cortex, Primary somatosensory cortex, barrel field, Secondary motor cortex

**TABLE 3:** The distribution of brain regions that innervate glutamatergic neurons in MVN.^39^

Brain regions	Nucleus
Medulla	Paramedian raphe nucleus
Pons	Dorsal cochlear nucleus, Superficial glial zone of the cochlear nuclei
Cerebellum	Central lobule, Crus 1 of the ansiform lobule, Culmen, Declive, Nodulus, Para‐flocculus, Paramedian lobule, Pyramus, Simple lobule, Uvula
Forebrain	Hippocampus, Main olfactory bulb, Somatomotor areas, Visual areas

**FIGURE 2:**
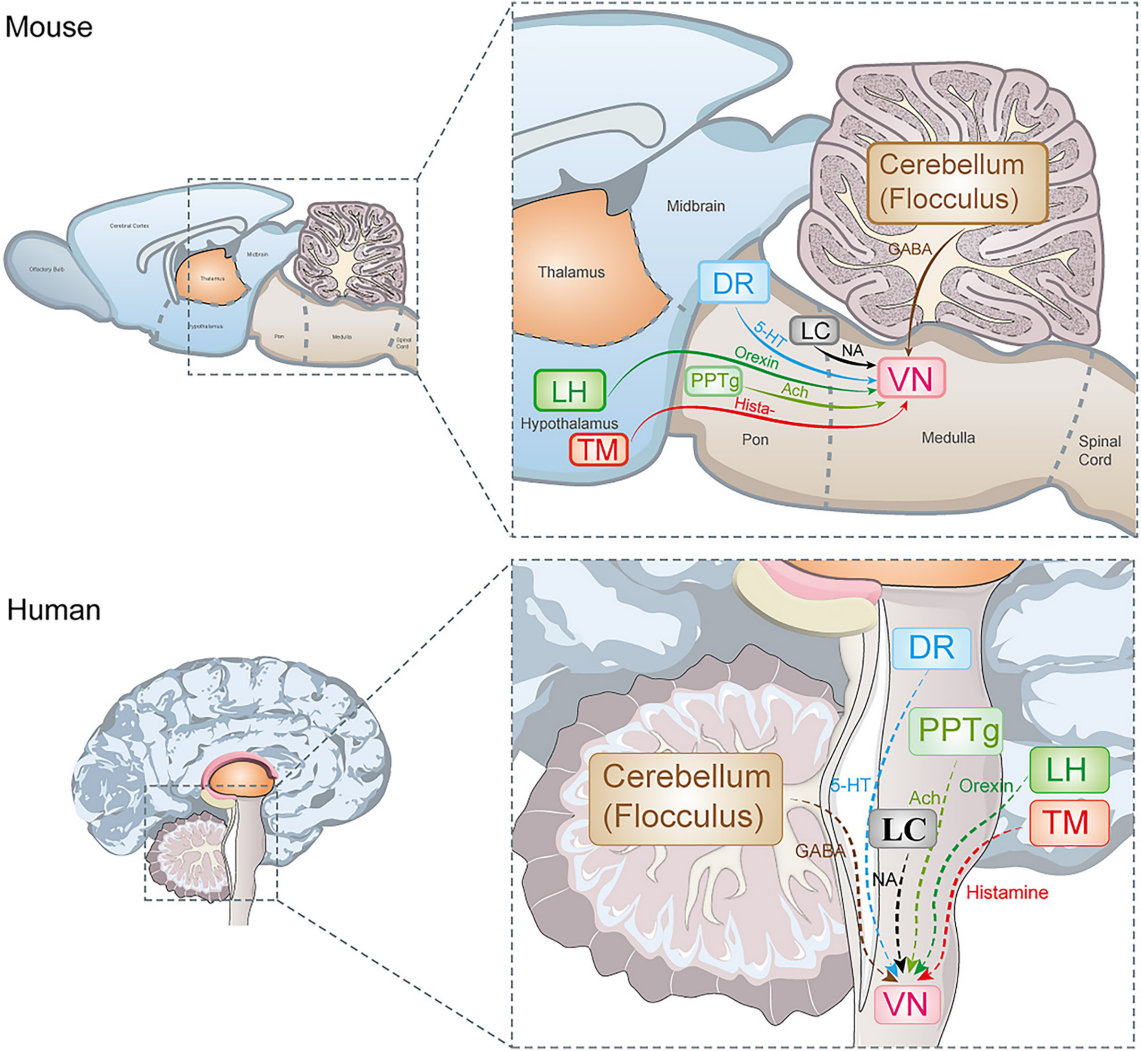
The structure of the hindbrain vestibular neural network in mice and humans.^47^, ^49^, ^50^, ^51^, ^52^, ^53^, ^54^ The VN integrate inputs from various brain regions and neurotransmitters to regulate vestibular compensation. These inputs include 5‐HT from the DR, histamine from the TM, Ach from the PPTg, orexin from the LH, NA from the LC, and GABA input mainly from the cerebellum, particularly the flocculus. The homeostasis of these neurotransmitters is essential for the complete function of the VN. The upper figure is of a mouse, and these data are derived from mice, indicated by the solid line. The lower figure is of a human, inferred from the results of the mouse, represented by the dashed line. DR, Dorsal raphe nucleus; LC, Locus coeruleus; LH, Lateral hypothalamic; PPTg, Pedunculopontine tegmental nucleus; TM, Tuberomammillary nucleus; VN, Vestibular Nuclei.

MVN receives inputs of neurotransmitters from different brain regions and integrates them to regulate postural control.^48^ The neurotransmitters include GABA, glutamate, histamine, 5‐hydroxytryptamine (5‐HT), noradrenaline, acetylcholine, and orexin (Figure 2).^47^, ^49^, ^50^, ^51^, ^52^, ^53^, ^54^ Here, we will discuss these neurotransmitters and their related brain regions.

### 6.1 Cerebellum and GABA

The cerebellum is the major regulator of body and eye movement, receiving input signals via mossy fibers and climbing fibers and innervating other brain regions through GABA release from Purkinje cells.^55^, ^56^, ^57^ The cerebellum has three lobes from the rostral to the caudal: the anterior, posterior, and flocculonodular lobes.^5^ Although the flocculonodular lobe is the smallest, it is highly connected with the vestibular nuclei and receives visual input.^58^, ^59^, ^60^ This region is one of the primary modulators of vestibular nuclei, and its deficit induces balance and gait disturbances.^61^, ^62^ The flocculus is located on the left and right border of the flocculonodular lobe, essential for the vestibulo‐ocular reflex, and involved in motor control.^63^, ^64^, ^65^ The flocculus receives input from the vestibular nuclei and projects to the magnocellular and parvocellular of MVN,^47^, ^66^, ^67^ this reciprocal innervation between the flocculus and MVN might contribute to vestibular compensation.^5^, ^8^, ^68^, ^69^, ^70^, ^71^, ^72^

**FIGURE 3:**
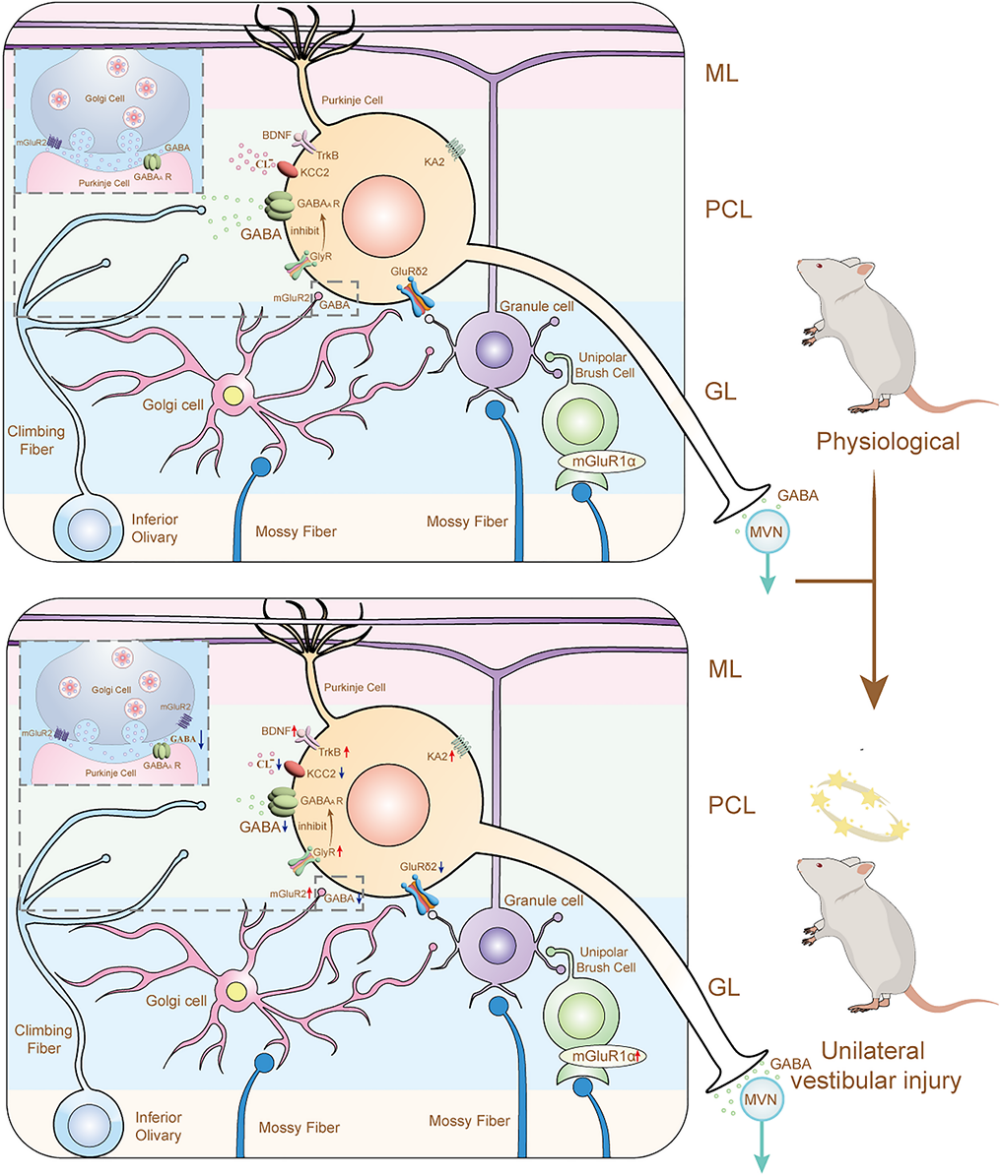
The mechanism of vestibular compensation in flocculus.^72^, ^73^, ^74^, ^75^, ^76^, ^77^, ^78^ After the unilateral vestibular injury, mGluR2 was up‐regulated on the Golgi cell, and mGluR1a was up‐regulated on the unipolar brush cells, in Purkinje cells, BDNF, TrkB, GlyR, and KA2 were up‐regulated, and KCC2, GluRδ2 were down‐regulated, this eventually leads to Purkinje cells excitably and regulates MVN to promote vestibular compensation. BDNF, Brain‐derived neurotrophic factor; ML, Molecular layer; GL, Granular layer; GlyR, Glycine receptor; KA2, kainate receptor subunit 2; KCC2, K(+)‐Cl(−) cotransporter 2; GluR, Glutamate receptor; MVN, medial vestibular nucleus; PCL, Purkinje cell layer; TrkB, Tropomysin related kinase B.

Figure 3 summarizes the results of previous molecular mechanism studies on vestibular compensation and flocculus.^72^, ^73^, ^74^, ^75^, ^76^, ^77^, ^78^ When the climbing fibers inputs to the flocculus are disrupted, vestibular compensation is delayed,^72^, ^79^, ^80^ suggesting the involvement of the flocculus in vestibular compensation. After the unilateral vestibular loss, a series of molecular changes in the flocculus are observed.^68^, ^76^ Brain‐derived neurotrophic factor (BDNF) upregulation may reduce the inhibitory effects of the flocculus by regulating inhibitory GABAergic synaptic transmission in floccular Purkinje cells and their terminals in the MVN.^73^ Glycine receptor (GlyR) mediates the downregulation of GABA_A_ receptor and regulates the Purkinje cell's excitability by inhibiting GABAergic effects,^75^ interestingly, the β subunit of GlyR was significantly increased from 8 hours to 3 days^81^ in MVN and the flocculus, which might activate ipsilateral MVN neurons via disinhibition. Protein Kinase C (PKC) isoforms display an asymmetric distribution in Purkinje cells after Unilateral labyrinthectomy (UL) and the Purkinje cells LTD was dependent on PKC activity. The recovery of PKC expression was synchronized with the disappearance of spontaneous nystagmus in rats,^74^ suggesting the importance of PKC in vestibular compensation.

Metabotropic glutamate receptors (mGluR) and ionotropic glutamate receptors also show significant changes during vestibular compensation. Ipsilateral flocculus mGluR2 is enhanced on the 1st day after UL, while mGluR7 remains unchanged.^59^ mGluR2 is present in the granular layer, and the presynaptic mGluR in the Golgi cell may be mGluR2, which further inhibits the inhibitory effect of the Golgi cell.^77^ The increase of mGluR2 in the ipsilateral flocculus eventually enhances long‐term depression (LTD) in the flocculus, resulting in reduced inhibition of MVN.^59^ The expression of mGluR1α in unipolar brush cells increases 1 h after the UL,^5^ upregulation of its expression enables unipolar brush cells to amplify upstream signals and transmit to granule cells. Ionotropic glutamate receptor KA2 increases at 6 hours, and reduces the inhibition of MVN.^78^ Downregulation of GluRδ2 receptors at the synapses of parallel fibers onto Purkinje cells also affects Purkinje cells regulation of MVN.^76^

Besides the flocculus, the vestibulo‐cerebellum also includes vermis, nodules/uvula (NU),^82^ essential for static posture and eye movements. These regions have direct reciprocal projection with MVN neurons,^39^, ^83^, ^84^, ^85^, ^86^ and the Purkinje cells of NU are also innervated by vestibular ganglion neurons,^72^, ^87^ and tuned to vestibular translation stimuli via mossy and climbing fibers.^88^, ^89^ In patients with vestibular neuritis, the local gray matter volume of the vermis decreased.^90^ After the unilateral vestibular deficit, the glucose metabolism of the bilateral vermis was up‐regulated.^91^ The expression of noradrenaline (NA) was up‐regulated in the vermis of UL rats.^92^ These results suggest that the vermis may regulate vestibular compensation.

Studies of vestibular compensation have so far focused on the regulation of MVN by flocculus, although the vestibulo‐cerebellum has long been thought to be related to MVN and to control motor balance, there have been few relevant studies.

### 6.2 Tuberomammillary nucleus (TM) and histamine

Histamine is crucial for arousal and cognitive functions including learning and memory.^93^ The TM in the posterior hypothalamus is the only source of histamine neurons in the CNS, modulating the target cells through histamine receptors H1, H2 and H3, activating Gq, Gs and Gi, respectively.^49^, ^50^, ^94^, ^95^ Some of the agonists and antagonists of these receptors are drugs for treating vestibular disorders, including unilateral vestibular damage.^49^ Unilateral vestibular injury reduces histamine staining of MVN and TM,^49^ and the expression of histidine decarboxylase mRNA within the ipsilateral TM is upregulated, which might result from the direct projection from the vestibular nucleus to the posterior hypothalamus, and the upregulation of histidine decarboxylase might result in the rebalancing of the bilateral MVN via the projection from TM to MVN.^96^, ^97^ H1 receptor is expressed in GABAergic neurons in MVN, promoting the vestibular compensation via asymmetric activation of the commissural inhibitory system.^98^ H3 receptor is an auto‐receptor on histaminergic and other types of terminals, its antagonists can also promote vestibular compensation,^99^, ^100^ for example, betahistine promotes vestibular compensation by acting on presynaptic histamine H3 and postsynaptic histamine H1 receptor.^49^ However, the mechanism of its function in vestibular compensation is largely unknown.

### 6.3 Dorsal raphe nucleus and 5‐HT

5‐HT, also known as serotonin, is released by the dorsal raphe nucleus and regulates vestibular nuclei.^51^, ^101^ 5‐HT receptors are all G protein‐coupled receptors, some of these receptors including 5‐HT1A, 5‐HT1B, 5HT2 and 5HT7, were expressed in all subregions of the vestibular nucleus.^102^, ^103^, ^104^, ^105^, ^106^ The 5‐HT1A and 5‐HT1B receptors inhibit neuronal activity by suppressing adenylate cyclase and cyclic adenosine monophosphate (cAMP), and activate the Mitogen‐activated protein kinase (MAPK) signaling pathway. The 5‐HT2A receptor activates phospholipase C and PKC, and influences the MAPK pathway. Contrary to the 5‐HT1 receptors, the 5‐HT7 receptor activates neurons.^107^ Studies have shown that 5‐HT plays a crucial role in regulating the vestibulo‐ocular reflex, which influences the eye movements of both awake and sleeping rats.^108^, ^109^, ^110^ This effect might be mediated by 5‐HT1A.^111^ During vestibular compensation, 5‐HT level increases in ipsilateral MVN,^101^, ^112^ and selective 5‐HT reuptake inhibitors (SSRIs) have been clinically applied to treat vertigo with or without psychiatric comorbidities.^113^, ^114^, ^115^ The patients of Meniere's disease with or without anxiety and depression can all be treated after giving SSRIs, suggesting that 5‐HT may act directly on the vestibular system and treat Meniere's disease.^113^, ^114^ However, how 5‐HT transmission is involved in vestibular compensation is poorly understood.

### 6.4 Locus coeruleus (LC) and NA

LC in the pons of the brainstem is the main source of NA in the brain.^52^, ^116^, ^117^ LC plays an important role in controlling balance and motor functions, and its damage can lead to mild to moderate ataxia, often leaning towards the side of the lesion.^118^ NA is also involved in both the vestibulospinal reflex and vestibulo‐ocular reflex, alterations in its levels can result in the deterioration of normal vestibular function.^119^ Retrograde tracing studies indicate a direct noradrenergic projection from the LC to the MVN.^120^ Impairment of this pathway seriously affects vestibular function and involves sensory mismatch during vertigo and motion sickness.^121^ Most MVN neurons are excited by NA via α1 and β receptors, while 82% of GABAergic neurons in MVN are activated by NA via α2 receptors and a few β receptors.^119^, ^122^, ^123^ During vestibular compensation, the expression of FOS protein increased significantly in LC, and noradrenergic neurons increased NA synthesis.^92^, ^124^, ^125^ However, injecting norepinephrine agonists into the ventricles result in decompensation behavioral effects.^126^ Therefore, the local effect of NA in each side of MVN needs further investigation.

### 6.5 Pedunculopontine tegmental nucleus and acetylcholine

Cholinergic neurons in the brain are located in the basal forebrain and brainstem,^127^ controlling vestibular function and processing sensory input information.^128^ The main group of acetylcholine neurons in the brainstem is located in the pedunculopontine tegmental nucleus (PPTg).^53^ They exert their influence by directly projecting to glutamatergic and GABAergic neurons within the vestibular nucleus.^38^, ^39^ Despite the lack of conclusive data, PPTg is most likely the source of acetylcholine for MVN.^129^ Electrophysiological studies in vitro have shown that acetylcholine, physostigmine, acetylcholinesterase inhibitor, or muscarinic agonists excite MVN neurons, and these effects are mediated by muscarinic receptors, which are antagonized by atropine and scopolamine.^130^, ^131^, ^132^, ^133^ After bilateral vestibular loss, acetylcholine neurons in PPTg are significantly activated.^129^ Local injection of acetylcholine or muscarinic receptor agonists into the vestibular nucleus resulted in postural deficits similar to those after unilateral vestibular injury.^134^, ^135^ The downstream effects of cholinergic neurons can be excitatory or inhibitory according to the type of muscarinic acetylcholine receptors the cell expresses.^136^ It also has been reported that knockout of the α9 subunit of the nicotinic acetylcholine receptor inhibits the recovery of nystagmus and vestibular compensation.^137^ However, the spectrum of acetylcholine receptor expression in MVN neurons remains largely unknown, especially the muscarine types of acetylcholine receptors.

### 6.6 Lateral hypothalamic and orexin

Orexin is synthesized by neurons in the lateral hypothalamus (LH),^138^, ^139^, ^140^ regulating feeding, energy homeostasis, sleep/wake cycle, and somatic motor control.^54^, ^141^ LH orexin neurons project to and release orexin to MVN, and orexin 1 (OX1R) and 2 (OX2R) receptors are expressed in MVN.^142^, ^143^ OX1R is coupled exclusively to the Gq subclass of heterotrimeric G‐proteins, whereas OX2R can couple to Gq and Gi/Go.^144^ Orexin excites the GABA neurons via co‐activation of OX1R and OX2R.^145^, ^146^ After the bilateral vestibular injury, orexin labelling neurons and the orexin expression in LH significantly increase for 3 days, which might result from direct projection from MVN to LH.^86^ The intracerebroventricular injection of the OX1R antagonist induces vestibular deficit behaviors, suggesting the importance of orexin in vestibular function.^147^ However, more research is needed to investigate orexin signaling in the vestibular system.

## 7 VESTIBULAR CORTEX AND VESTIBULAR COMPENSATION

The vestibular system in the cortex is a dispersed network of several cortical brain regions, unlike the auditory or visual systems, which have their own dedicated primary cortex.^148^, ^149^, ^150^, ^151^ This distribution configuration of the cortical vestibular network, which offers resilience against cortical lesions like stroke and traumatic brain injury, may result from the integration of information from several sensory systems.^149^ However, whether this distributed cortical vestibular network could also contribute to the vestibular compensation after peripheral vestibular dysfunction is still unknown. This may be partly explained by the notable brain region differences between primates and other animal models, such as rodents.

**FIGURE 4:**
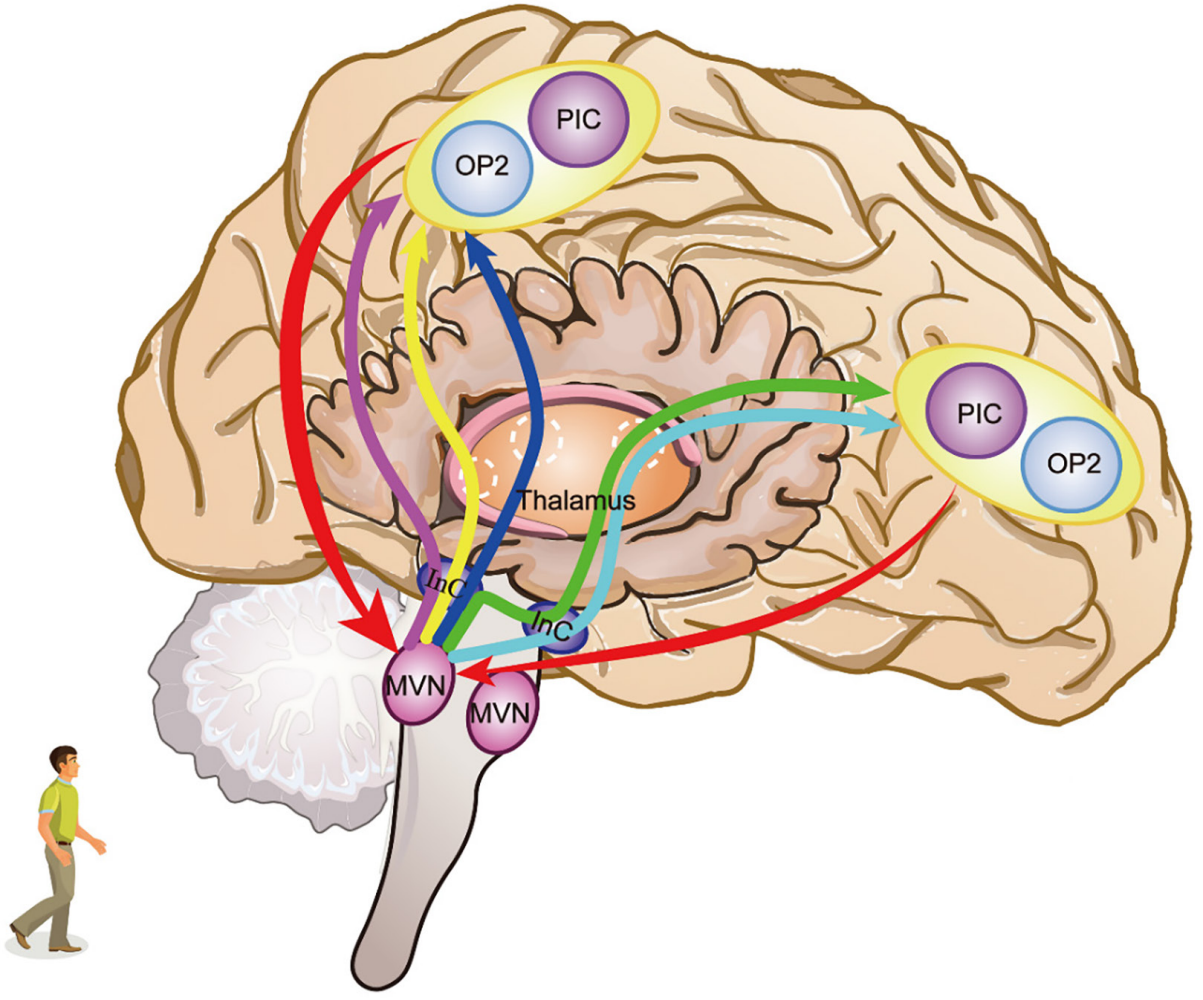
The connection between vestibular cortex and MVN in humans.^158^ Vestibular cortex projects directly to MVN, and through the vestibular‐thalamic‐cortex connection, MVN indirectly projects to the cortical vestibular network. InC, Interstitial nucleus of Cajal; MVN, Medial Vestibular Nucleus; OP2, Parietal operculum cortex 2; PIC, Posterior insular cortex.

The cortical vestibular network in human is composed of at least 10 cortical regions, including area 2v and 3a regions in the primary somatosensory cortex, area 7 of the parietal cortex, the premotor area, cingulate sulcus visual (CSv), ventral intraparietal area (VIP), parietal operculum cortex 2 (OP2)/parieto‐insular vestibular cortex (PIVC), posterior insular cortex (PIC)/visual posterior sylvian area (VPS), supplementary motor area (SMA) and human medial superior temporal area (hMST).^149^ Among these regions, OP2/PIVC and PIC/VPS that located in the Sylvian fissure are densely interconnected, and OP2/PIVC is considered as the core of the cortical vestibular network.^152^ Nevertheless, the rodents lack the OP2/PIVC homologous brain region; vestibular signals instead activate the primary sensory cortical cortex, infralimbic, and cingulate cortices in the frontal regions.^153^ This may occur via the MVN ascending pathway, which goes to the bilateral sensorimotor cortices after projecting to the ipsilateral ventral posterior (VP) and ventral anterior (VA) thalamus.^154^ This indirect ascending pathway of MVN to the cortex via the thalamus is also conserved in primates.^155^ In response, the OP2/PIVC in primates directly projects to the MVN,^148^, ^156^ regulating MVN‐dependent vestibular reflexes, including the vestibulo‐ocular, the vestibulo‐spinal, and the optokinetic reflexes (Figure 4).^157^, ^158^

In summary, the primate animal model will be crucial for the future investigation on the role of cortical vestibular network, especially OP2/PIVC, during vestibular compensation.

## 8 CLINICALLY SIGNIFICANT

Currently, vestibular suppressants and antiemetic medications are the predominant treatments for vertigo.^159^ Anticholinergics, antihistamines, and benzodiazepines are among the vestibular suppressants; 5‐HT3 receptor antagonists, dopamine antagonists, and some antihistamines are among the antiemetic medications.^159^, ^160^, ^161^ Through exercises, vestibular rehabilitation therapy facilitates the reorganization of the neural network by training the CNS and sensory system to accommodate vestibular impairment.^162^, ^163^ Understanding the vestibular neural network will facilitate the discovery of novel drug targets and rehabilitation strategies.^164^

## 9 FUTURE DIRECTIONS

The bilateral vestibular nuclei are the essential hub for vestibular compensation, the commissural inhibitory system between the bilateral vestibular nuclei is crucial for the activity balance in the two nuclei and functional recovery from unilateral vestibular damage. Recent advances in neural networks revealed several neurotransmitters, including glutamatergic, GABAergic, histaminergic, cholinergic, serotonergic, and adrenergic neurons are involved vestibular compensation, however, more research is needed to elucidate their mechanism in vestibular compensation, which will greatly facilitate developing new treatment strategies for vestibular system‐related diseases.

Why do patients with the same vertigo condition experience varying outcomes in rehabilitation and prognosis? Vertigo and other conditions can exacerbate each other, creating a vicious cycle that frequently results in diminished treatment effectiveness and unfavorable outcomes. For the challenges and poor prognoses associated with vertigo treatment, this review delves into the intricate regulatory mechanisms of the vestibular neural networks, offering a novel approach, establishing a neuropharmacological basis for clinical drug therapies and tailored rehabilitation strategies for vertigo patients. With a clear vision and innovative spirit, ‘Lead the way, carve a unique path.’

## AUTHOR CONTRIBUTIONS

SLZ, HYY and YSL conceived the project and developed its outline; JW, YJZ and HJY drafted the manuscript. JW, ZYJ, ET, ZQG and JYC participated in data acquisition and figure preparation. CJQ, HQJ, ZHZ, JQG, QL and SYS reviewed and revised the manuscript. All co‐authors contributed to the article and approved the final manuscript.

## FUNDING INFORMATION

This work was supported by grants from the National Key Research and Development Program of China (grant nos. 2023YFC2508002 & 2023YFC2508403& 2023YFC2508005), the National Natural Science Foundation of China (grant nos. 82371168 & 82171152), the Central Funds Guiding the Local Science and Technology Development (grant no. 20221ZDG020066), Key Clinical Specialty of Jiangxi Province (grant no. 202382), and the Hubei Provincial Key Research and Development Program (grant no. 2023BCB027).

## CONFLICT OF INTEREST STATEMENT

The authors declare no competing interests.

## Data Availability

Data sharing not applicable to this article as no datasets were generated or analysed during the current study.
